# Cultivated Oral Mucosa Epithelium in Ocular Surface Reconstruction in Aniridia Patients

**DOI:** 10.1155/2015/281870

**Published:** 2015-09-16

**Authors:** Dariusz Dobrowolski, Boguslawa Orzechowska-Wylegala, Bogumil Wowra, Ewa Wroblewska-Czajka, Maria Grolik, Krzysztof Szczubialka, Maria Nowakowska, Domenico Puzzolo, Edward A. Wylegala, Antonio Micali, Pasquale Aragona

**Affiliations:** ^1^Department of Ophthalmology, Ophthalmology Clinic, II School of Medicine with the Division of Dentistry in Zabrze, Medical University of Silesia, District Railway Hospital, Panewnicka 65 street, 40760 Katowice, Poland; ^2^Clinic of Maxillo-Cranio-Facial Surgery, Clinical Hospital, Medical University of Silesia, Francuska 20-24 street, 40027 Katowice, Poland; ^3^Department of Nanotechnology of Polymers and Biomaterials, Faculty of Chemistry, Jagiellonian University, Ingardena 3 street, 30060 Cracow, Poland; ^4^Department of Biomedical Sciences and Morphofunctional Imaging, University of Messina, 98 125 Messina, Italy; ^5^Department of Surgical Specialties, Section of Ophthalmology, University of Messina, Policlinico “G. Martino”, Gazzi, 98125 Messina, Italy

## Abstract

*Purpose*. Efficacy of cultivated oral mucosa epithelial transplantation (COMET) procedure in corneal epithelium restoration of aniridia patients. *Methods*. Study subjects were aniridia patients (13 patients; 17 eyes) with irregular, vascular conjunctival pannus involving visual axis who underwent autologous transplantation of cultivated epithelium. For the procedure oral mucosa epithelial cells were obtained from buccal mucosa with further enzymatic treatment. Suspension of single cells was seeded on previously prepared denuded amniotic membrane. Cultures were carried on culture dishes inserts in the presence of the inactivated with Mitomycin C monolayer of 3T3 fibroblasts. Cultures were carried for seven days. Stratified oral mucosa epithelium with its amniotic membrane carrier was transplanted on the surgically denuded corneal surface of aniridia patients with total or subtotal limbal stem cell deficiency. *Outcome Measures*. Corneal surface, epithelial regularity, and visual acuity improvement were evaluated. *Results*. At the end of the observation period, 76.4% of the eyes had regular transparent epithelium and 23.5% had developed epithelial defects or central corneal haze; in 88.2% of cases visual acuity had increased. VA range was from HM 0.05 before the surgery to HM up to 0.1 after surgery. *Conclusion*. Application of cultivated oral mucosa epithelium restores regular epithelium on the corneal surface with moderate improvement in quality of vision.

## 1. Introduction

During second decade of life, aniridic patients begin developing pathologic cellular exchange. Epithelial cells covering the cornea are removed by the surrounding conjunctival epithelium with its vascularized matrix [1]. This leads to a local disorder called limbal stem-cell deficiency (LSCD) [2]. Its stages lead to superficial conjunctival ingrowth, corneal haze, discomfort, and deep loss of visual acuity. Since the 1980s, the concept of limbal disease management has focused on limbal transplantation and removal of pathologic tissue followed by transplantation of autologous or allogeneic limbal tissue carried on the donor's sclera [3, 4]. Many studies have confirmed that delivery of healthy limbal tissue improves vision and decreases ocular discomfort [5–7].

Development of new culture techniques has changed the treatment of LSCD [8]. Transplantation became limited to cultured autologous cells, without additional tissues of the donor's sclera or conjunctiva [9]. Application of oral mucosa epithelium was proposed by Shigeru Kinoshita and was turned into clinical practice by Nakamura et al. [10]. Another new concept was use of oral mucosa epithelium in reconstruction of the ocular surface [11]. With a phenotype similar to the cornea, it allowed the possibility of autologous treatment in patients with bilateral ocular involvement [12].

Congenital aniridia is a rare disorder of autosomal-dominant or sporadic occurrence. It occurs with an incidence of slightly less than 1 : 100,000 to 1 : 60,000 in the general population. It manifests with low visual acuity and amblyopia early. Later it usually is accompanied by cataract and glaucoma. From the second decade of life, an additional factor influences visual quality: corneal haze caused by the conjunctivalization of the cornea surface [13].

In aniridia patients, signs of LSCD are ocular discomfort, superficial vascular conjunctival pannus, and decreased visual acuity. Severity is mild; therefore, for several years those patients were disqualified from any surgery. Cultivated oral mucosa epithelium transplantation (COMET) offers minimal and effective surgical approach for this group of patients.

## 2. Materials and Methods

The experimental procedure was performed under the tenets of the Declaration of Helsinki. Signed written informed consent was obtained from all patients before the procedure began.

Oral mucosa epithelium specimens (3–5 mm^2^) were collected under local anesthesia and decontaminated with 5% povidone-iodine solution. Biopsies were taken from the inferior buccal mucosa; no sutures were applied. The tissue was transferred immediately to corneal storage medium at 4°C and then taken to the laboratory.

The tissue specimen was treated with Dispase II for 1 hour, after which it was trypsinized with 1% trypsin/0.01% EDTA mixture for 10 min in 37°C to obtain a single cell suspension. Epithelial cells were prepared for seeding with a density of 4  ×  10^4^ cells per 1 mL (Cell counter, Coulter Z1, Miami, USA).

Culture media and chemicals were purchased from Sigma (Germany). The reagents for immunostaining were obtained from Santa Cruz Biotechnology Inc. (USA). The epithelial culture was carried in the presence of 3T3 fibroblasts, a source of growth factors. One week prior to specimen collection, six-well culture dishes (Becton Dickinson, USA) were prepared. Bottoms of the plates were covered with a monolayer of 3T3 fibroblasts (ATCC, USA). The 3T3 cells were cultivated in Dulbecco Modified Eagle's Medium (DMEM) with 10% bovine serum and a 100 *μ*g/mL penicillin and streptomycin mixture. From the fifth to seventh day of culture, when the monolayer was reached, fibroblasts were inactivated by incubation in DMEM containing 2 *μ*g/mL of Mitomycin C for 2 h.

The carrier for the oral mucosa epithelial sheet was the amniotic membrane located on the dish insert over the monolayer of fibroblasts. Amniotic membrane carriers were obtained from the Homograft Tissue Bank in Zabrze, Poland. Amniotic membrane samples were washed from the cryoprotective medium with Phosphate Buffered Saline (PBS), and the amniotic epithelium was removed gently with a culture scraper. Denuded amniotic membrane slides (15 × 15 mm) on nitrocellulose paper were put over the layer of fibroblasts in the culture plates.

**Figure 1:**
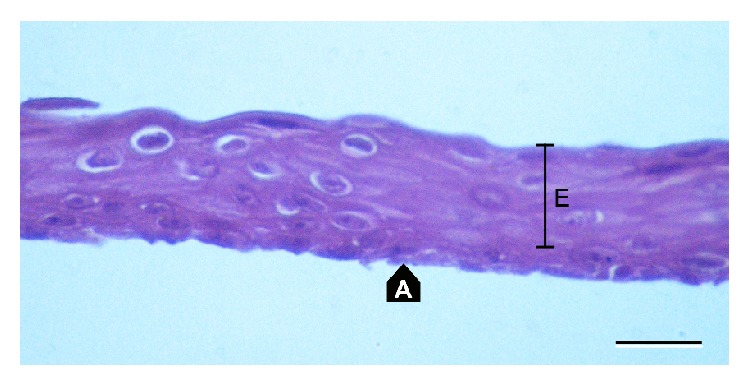
Cultivated oral epithelium (E) on the amniotic membrane (A) prior to transplantation. H&E stain. Scale bar: 20 *μ*m.

Single oral mucosa cells were seeded on denuded amniotic membrane spread on nitrocellulose paper lying on the inserts over the fibroblast monolayer. Cultures were carried out in standard conditions of 37°C and a humidified atmosphere of 5% CO_2_ and 95% air. The medium was a supplemented Dulbecco Modified Eagle's Medium (DMEM) with HAM F12 mixture with 10% of serum (bovine serum in first 12 experiments, autologous serum in the next 5 cultures), 0.5% dimethyl sulfoxide (DMSO), 10 ng/mL mouse Epidermal Growth Factor (EGF), 5 *μ*g/mL bovine insulin, 0.1 nM cholera toxin, 0.18 mM adenine, 2 nM triiodothyronine, 4 mM L-glutamine, 0.4 mg/mL hydrocortisone, and 100 *μ*g/mL penicillin and streptomycin mixture. The culture medium was changed every 48 h. Cultures were carried out on at least 2 amniotic membranes for each patient. On the seventh day of culture, the epithelial growth stage was evaluated under light microscope and small specimens of each membrane (Figure 1) were stained with hematoxylin-eosin (H-E). Amniotic membranes (A in Figure 1) covered completely with multilayer epithelium (E in Figure 1) were suitable for a graft. Cultivation over 7 days caused loss of superficial cells of the proliferating epithelium to the medium. At the time of microscopic qualification, airlifting was done. Cells were airlifted with a minimal amount of culture medium for 1 h.

One day before the transplantation, immunostaining for cytokeratin 4, cytokeratin 13, protein p63, and connexin 43 was performed to confirm the origin of the epithelium and identify the presence of low differentiated cells. Both cytokeratins 4 and 13 were described by Ang et al. as characteristic for oral mucosa epithelial cells, their presence confirming expected cellular structure of the graft just before application [14]. Protein p63 (transcription factor) plays an important role in epithelial cells proliferation and differentiation, in cultured cells it shows cellular potential for epithelial renewal [15]. Gap-junction protein connexin 43 is present in the basal epithelial layer, and positive staining confirms proper architecture of cultured epithelial layers [16]. Hematoxylin-eosin (H-E) staining was used in histological analysis of epithelial layer regularity and thickness. Regular structure of intact epithelium on amniotic membrane presented on Figure 1 was expected by researchers. Only such grafts were qualified in surgery.

Study subjects were 13 aniridia patients (17 eyes) suffering from LSCD with central corneal involvement. Vascular conjunctival pannus involved the entire limbal area (total LSCD: 14 eyes) or at least 9 clock-hours of the limbus with central involvement (subtotal LSCD: 3 eyes). The main indication for the study was presence of semitransparent conjunctival pannus covering the cornea over the pupil. It caused decreased quality of vision even in cases of subtotal limbal involvement. The patients were both oral mucosa epithelium donors and recipients of the cultured epithelium. There were 3 men and 10 women. Ages ranged from 16 to 54 with an average of 31.1 ± 11.5 years. In 4 cases, surgery was bilateral and sequential (3–6 months between each surgery). Visual acuity before intervention ranged from HM to 0.05. Inclusion criteria were congenital aniridia, conjunctivalization of the corneal surface with neovascularization (with or without corneal scarring) and at least 6 months from last eye surgery. In addition, the study required healthy oral mucosa, no smoking for at least 7 days before taking a sample of epithelium, and regular teeth brushing (twice a day). Exclusion criteria were acute systemic infections, acute eye infection during the last 6 months, neoplasm history, pregnancy, and mental disorders. In addition patients with dense cataract coexisting corneal disorders were excluded.

Surgery was performed under topical anesthesia (Alcaine, Alcon, USA). A 360-degree peritomy was done, followed by conjunctival suturing at 1-2 mm behind the limbal area with single 10-0 nylon sutures. The conjunctival pannus was removed gently with a spatula or crescent knife. If necessary, fibrotic tissue spread under the conjunctival pannus was removed with gentle keratectomy to prepare a smooth corneal surface. Then, round, 14 mm diameter trephined amniotic carriers with oral mucosa epithelial cells were transplanted onto the denuded corneas. Peripheral, continuous 10-0 nylon suture was used to stabilize the graft. The entire cornea was covered, and suture was placed in the corneolimbal interface. Then, the graft was covered by a bandage contact lens.

After the procedure, levofloxacin (Santen, Finland) and preservative-free dexamethasone eye drops (Thea Laboratories, Paris, France) were administered 5 times a day for 2 weeks. Then, dexamethasone alone was applied 3 times a day for 6 weeks. All subjects received preservative-free lubricants every 2 hours, and in cases of epithelial defects or erosions, autologous serum drops were administered. On each control visit, stabilization of the corneal surface was evaluated with topical 10% fluorescein staining. Lack of epithelial defects (negative fluorescein staining) with regular, stratified epithelium or no more than 3 clock-hours of peripheral conjunctival neovascularization was classified as a successful case (coded as +++); partial success consisted of punctate epithelial defects or peripheral conjunctival neovascularization up to 9 clock-hours without pupil area involvement (coded as ++); and failure consisted of vascularization of the pupil area, persistent nonhealing epithelial defects or deep stromal vascularization (coded as +). Whether LSCD occurrence was total or subtotal made no differences in surgical management or postoperative treatment regarding 360-degree peritomy and removal of all fibrotic tissue or epithelia from the corneal surface.

## 3. Results

Local complications were not seen after oral mucosa donation. Transplanted specimens showed regular, stratified epithelium of 4–9 layers of cells. Epithelium origin was confirmed by previous immunostaining, as described in the culture procedure section.

**Figure 2:**
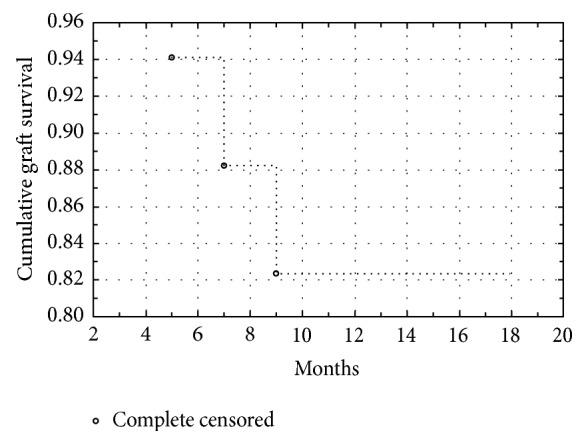
Cumulative graft survival: Kaplan-Meier curve for COMET in aniridia.

**Figure 3:**
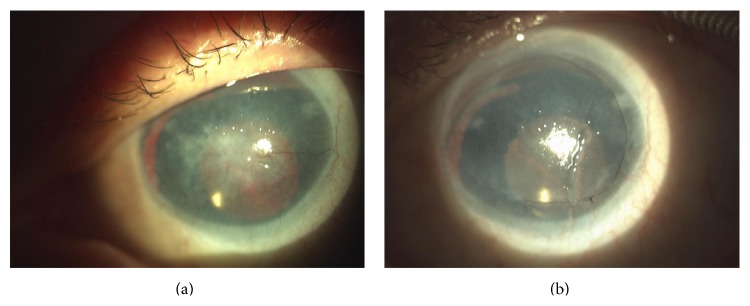
(a) and (b) Aniridia patient, 49-year-old man, who underwent cataract surgery 5 years before COMET. Preoperative view with central fibrovascular scar on the left; on the right, 6 months after surgery, VA improvement from CF to 0.1 with central cornea covered by regular and smooth epithelium.

Over the observation period of 12–18 months, 13 operated eyes showed stable epithelium; however, peripheral vascular irregularities were present in all of them.  Table 1 shows detailed patient data. These corneas were not stained by fluorescein solution (qualified as +++); 4 eyes suffered various epithelial defects, from single local defects (++) to large areas of denuded cornea (+) with peripheral conjunctival ingrowth. At the end of the observation period, 3 eyes developed stromal scarring, conjunctival vascularization, or stromal vascularization. All 3 of these eyes were qualified as graft failure. In successful cases, the epithelium was smooth and regular (qualified as +++ and ++).  Figure 2 presents the graft survival rate on a Kaplan-Meier curve. The first 9 postoperative months were critical to the final results. Occurrence of epithelial defect was the indication for autologous serum eye drops. Despite careful treatment, 3 failed cases developed after 6 months of progressive invasion of the central cornea by vascular conjunctival tissue. In other cases, the peripheral area was characterized by irregularities in the vascular network, with peripheral ingrowth of new vessels without central area involvement. Comparison of surfaces before and after surgery (Figures 3(a) and 3(b)) shows improvement of corneal transparency in the central area and irregular distribution of vessels in the limbal area. Inflammatory response, defined as hyperemia and neovascularization at the donor-host interface, was moderate and easily controlled with a steroid agent. Limited vascular ingrowth in eyes with stable autologous oral epithelium confirms that COMET with its autologous origin limits inflammatory response. There is also no immune reaction characteristic for allotransplantation in, for example, keratolimbal allografts (KLAL). Excluding the failed grafts, patients noted improvement in their quality of vision, as well as decreased ocular discomfort. Postoperative occurrence of epithelial defects did not interfere with visual acuity and were treated with autologous serum drops 7 times a day. Four eyes with epithelial defects received persistent therapy; in three others (graded as +++) therapy was temporary for up to four weeks. Visual acuity improvement was noted in 88.2% of cases. It ranged from HM up to 0.1. As shown in Figure 3 main benefit of COMET was restoring of transparent epithelium without pathologic vessels on the corneal surface. This 49-year-old, single-eyed man developed conjunctival pannus after cataract surgery performed 5 years ago. Improvement to 0.1 restored his ability to read; he pointed it as a main benefit of surgery. Despite moderate improvement of VA the majority of patients were satisfied after surgery.

## 4. Discussion

LSCD is a difficult therapeutic challenge in patients with congenital aniridia. This condition is mild in severity and is usually accompanied by amblyopia, cataracts, and corneal haze caused by conjunctival pannus. It is believed that progressive conjunctivalization with age is connected to congenital impairment of pluripotent cells located in the hem.

In 2003, Nakamura and Kinoshita proposed a cultivation method of oral epithelium to restore ocular epithelial surface based on experiments on rabbits [17]. Several benefits made the procedure very interesting. Damaged corneas covered by autologous epithelium have a lower risk of immunologic reaction. Oral epithelium transplanted onto the new locus can maintain its proliferative potential without developing keratinization. In addition, low differentiation and quick cellular turnover result in regular stratified epithelium.

An important factor of this method is the cellular phenotype of oral epithelium. The phenotype is similar to the corneal epithelium phenotype, making this procedure safe and easy to apply. Allografts often require systemic or local immunosuppressive agents administered for long periods postoperatively. Autografts do not have such requirements, as the induction of neovascularization is lower if the tissue comes from the same patient [18].

As described by Hata et al., oral epithelium culture is very effective for recipients, as these cells have a low stage of differentiation combined with fast cellular turnover [19]. To obtain a regular epithelial layer, only a few days of culturing are needed. In addition, these features are prolonged for the postoperative period. In long-term observation, it improves stability of the transplanted epithelium. In our patients, the surface epithelium in successful cases remained stable with temporary epithelial disorders. In local defects, autologous serum drops were administered to improve epithelial growth. For large defects autologous serum or bandage contact lenses were ineffective. Such patients in our study developed secondary conjunctival ingrowth leading to failure of the procedure.

COMET is widely proposed in Stevens-Johnson Syndrome (SJS), ocular cicatricial pemphigoid (OCP) and ocular burns. However, in those cases many patients require 2-step surgical approach to restore vision due to accompanied stromal damage. Sotozono et al. describes such cases showing that long-term visual improvement is possible even with high rate of postoperative epithelial defects (40%) [20]. In acute cases of SJS or OCP damage of the ocular surface and inflammation grade is high. Authors have only one case of aniridia and a few cases with slight ocular surface damage. In those cases visual improvement is significantly high. In aniridic patients ocular surface disease is limited to the epithelial surface and subepithelial fibrosis. There is no severe inflammation or destruction of surrounding tissues. In our group there was also no need of stromal procedures of lamellar or penetrating keratoplasty. Final vision remained low; however, main benefit was stable epithelial surface with only 3 failed grafts and 4 patients with epithelial defects (3 of them were temporary). If compared to severe cases of ocular surface disease in aniridia there was no indication to administration of systemic steroids.

Historically, therapeutic treatment has included Scheffer Tseng's approach of removing the corneal pannus combined with lamellar keratoplasty or penetrating keratoplasty combined with limbal allotransplantation. However, allotransplants require subsequent systemic immunosuppression, which can interfere with the patient's general health. Complicity and difficulty of the technique resulted in the need to transplant only those epithelial cells involved in the pathologic process. COMET offered the opportunity to focus on the affected area of the corneal surface.

Tsubota et al. describing keratolimbal allografts shows high risk of immune adverse reactions (graft rejection), infections, and systemic disorders associated with immunosuppressive agents administered for many postoperative months [21]. In his study stable ocular surface in achieved in 51% of the cases. If we compare improvements in vision, allografts limit the quality of vision [22]. Risk of rejection and revascularization result in loss of visual acuity in long-term observations. The Kaplan-Meier curves for the allograft approach show continuous reduction of success rate in the long term. Daya et al. reports results of allogeneic cultured corneal epithelium transplantation with success rate of 70% [23]. It indicates that limitation of surgical approach to epithelial surface only increases success rate. Nishida et al. report 100% success rate after COMET; however, in other studies authors achieve stabilization of corneal surface in 66.7% or even 57.5% [24–26]. The autologous approach with oral epithelium transplantation seems to cause less immunization, less inflammatory response, and less vascular ingrowth. These benefits should increase the success rate in bilateral limbal stem-cell insufficiency present in aniridia.

There were not many differences between culture procedures for the oral and corneal epithelium transplantation techniques. Amniotic membrane substrate as a cellular carrier and the source of basement membrane is widely applied [27, 28]. Its efficacy is confirmed in papers on cultured epithelial transplantation. Amniotic membrane makes the application of oral epithelial sheets safe and easy. In mild cases with partial limbal insufficiency in aniridia, transplantation limited to the application of amniotic membrane can support the corneal epithelium for a long period of time. Coculture with 3T3 fibroblasts results in fast proliferation and maintains low differentiation of the oral epithelium. This parameter is crucial for graft survival, despite fast cellular turnover. Another advantage is the almost unlimited source of tissue, which can be collected easily. The regenerative potential of oral epithelium is high. During cultivation, loss of superficial epithelial cells to the media was observed after seven days. In our opinion, a culture period of one week is sufficient to reach stable oral epithelial multilayers.

In 2003, Nakamura et al. described the first oral epithelium transplantations in humans with corneas severely damaged by Stevens-Johnson Syndrome (SJS) and severe ocular burns. Results were promising, and oral epithelium became a substitute for corneal epithelium with satisfactory corneal transparency and reduction of ocular discomfort. Nakamura et al. later described 17 patients treated with the COMET procedure with a mean follow-up of 55 months [29]. All eyes manifested revascularization of the surface with various stages of severity. The most stable epithelia were observed within six months after surgery. Visual acuity improvement was observed in 95% of patients after surgery. After 36 months, only 53% of eyes noted improved visual acuity. The authors concluded that despite the decrease in vision quality, the reconjunctivalization was less severe than that before surgery. In failed cases presented in the paper, conjunctival invasion was not severe; however, involvement of visual axis caused failure of the entire treatment.

Another paper on COMET by Ang et al. described stabilization of the ocular surface without major complications in all investigated eyes for at least 12 months after surgery [14]. Graft survival was limited in COMET; however, scarification of the ocular surface was much less severe compared with untreated patients. In addition, combining COMET with penetrating keratoplasty led to improved ocular surface and decreased risk of PK failure [30].

Satake presented the results of 40 eyes after COMET. Procedure were performed on SJS, cicatricial pemphigoid, and ocular burn patients [26]. Results showed the fast decline of graft survival in the first six months after the procedures with subsequent stabilization. Outcomes depended on the severity of ocular involvement; patients with severe vascularization and damage to the cornea surface were at greater risk of early graft failure than those with less severe symptoms. Similarly, poor primary visual acuity resulted in a worse outcome for those in the aniridia group. However, analysis has not been performed on patients with congenital aniridia. According to our study, graft survival and epithelium regularity are much better in aniridia patients.

Visual acuity in patients with aniridia depends on the optical transparency of both the cornea and the lens. In 75% of patients, cataracts occurred next to the corneal pannus, which greatly affected visual acuity. Therefore, in our opinion, visual acuity is a poor success parameter of transplant-cultured oral mucosal epithelium. In the success group graded +++, there were eyes with no visual acuity improvement despite a stable surface, showing that other factors, such as foveal hypoplasia, optic nerve lesions, or cataracts, were disturbing vision. In our study, the criterion for success was restoration of the corneal epithelium, although it is known that the most important criterion for patients is visual acuity. Increased corneal vascularization with patient age is associated with epithelial cell dysfunction, but the exact cause has not been documented clearly yet. Some authors make effective use of anti-VEGF, but the literature does not describe the use of anti-VEGF in corneal neovascularization.

Application of COMET was successful in aniridia patients. The visual acuity improvement rate for aniridia cases was lower than that for SJS, ocular cicatricial pemphigoid, or ocular burn cases. While actual visual acuity improvement was lower, the main benefit was improvement of epithelial regularity. Chen et al. investigated cell survival in COMET patients [31]. The majority of recipients in the follow-up had oral mucosa-derived cells The present study has confirmed the usefulness of the procedure in cases of mild limbal insufficiencies, such as in aniridia patients. In conclusion, COMET is a very useful surgical procedure for patients with congenital aniridia, as it delivers benefits with minimal harm. Further studies are needed to compare epithelium restoring procedures including etiology of limbal stem deficiency.

## Figures and Tables

**Table 1:** Overview of aniridia patients data.

Case	Age	Gender	Eye	BCVA pre	BCVA post	Epithelial regularity	Follow-up (m.)	Treatment
1	28	F	R	CF	0.05	+++	18	Routine
2	28	F	L	0.05	0.1	+++	12	Routine
3	27	F	R	0.02	0.1	+++	14	Routine
4	54	F	R	HM	CF	+	13	Routine, PSED, DEX
5	26	F	L	0.02	0.05	+++	18	Routine
6	29	F	R	0.05	0.05	+++	18	Routine
7	27	F	L	0.01	0.03	+	17	Routine, PSED, DEX
8	18	F	L	HM	0.1	+++	18	Routine
9	18	F	R	HM	0.05	+++	14	Routine
10	49	M	L	CF	0.1	+++	18	Routine
11	40	F	R	CF	0.05	+++	15	Routine, TSED
12	23	M	L	HM	CF	+++	18	Routine, TSED
13	38	F	L	0.01	0.02	+++	18	Routine
14	38	F	R	HM	0.05	++	15	Routine, PSED
15	49	M	L	CF	0.1	+++	12	Routine
16	16	F	L	HM	HM	+++	18	Routine, TSED
17	22	F	R	HM	0.05	+	16	Routine, PSED, DEX

Fluorescein staining: absent (+++), local defects (++), and present (+).

CF: counting fingers and HM: hand movements.

Routine treatment: Levofloxacin and dexamethasone eye drops 5 times a day for a 2-week period and then only dexamethasone 3 times a day for the next 6 weeks.

Additional treatment: PSED: permanent serum eye drops, TSED: temporary serum eye drops, and DEX: additional dexamethasone administration over 2nd month from surgery.

## Summary Statement

COMET procedure is a minimal invasive treatment, which enable exchanging irregular corneal epithelium for regular stratified oral mucosa epithelium. Aniridia causes huge discomfort; COMET not only restores stable ocular surface but also improves quality of life. Another advantage is simple surgical approach, autologous transplantation, and nonaggressive postoperative treatment. Visual acuity improvement is more efficient for near than for far distance; the majority of patients were able to read again.
